# Teacher Training Effectiveness in Self-Regulation in Virtual Environments

**DOI:** 10.3389/fpsyg.2022.776806

**Published:** 2022-03-28

**Authors:** María Consuelo Sáiz-Manzanares, Leandro S. Almeida, Luis J. Martín-Antón, Miguel A. Carbonero, Juan A. Valdivieso-Burón

**Affiliations:** ^1^Departamento de Ciencias de la Salud, Facultad de Ciencias de la Salud, Research Group DATAHES, Universidad de Burgos, Burgos, Spain; ^2^Instituto de Educação, Research Group CIEd, Universidade do Minho, Braga, Portugal; ^3^Department of Psychology, Excellence Research Group GR179 Educational Psychology, University of Valladolid, Valladolid, Spain

**Keywords:** self-regulated learning, gamification, learning management systems, virtual environments, teacher training, higher education

## Abstract

Higher education in the 21st century faces the challenge of changing the way in which knowledge is conveyed and how teachers and students interact in the teaching-learning process. The current pandemic caused by SARS-CoV-2 has hastened the need to face up to this challenge and has furthered the need to approach the issue from the perspective of digitalisation. To achieve this, it is necessary to design training programmes geared towards teaching staff and which address both the use of technology and instructional design aimed at promoting the development of self-regulated learning (SRL) and automatic feedback systems. In this study, work was carried out with 23 teachers (8 inexperienced and 15 experienced teachers) in a training programme conducted through Moodle. The aims were: (1) to test whether there were any significant differences between the behaviour patterns of new teachers compared to experienced teachers, (2) to determine whether clusters of behaviour patterns corresponded to the type of teacher and (3) to ascertain whether the level of teacher satisfaction with the training activity in digital teaching will depend on the type of teacher. A quantitative as well as a qualitative design was applied. Differences were found in the behaviour patterns in the training activities for the development of rubrics and use of learning analytics systems in virtual learning environments. It was also found that the type of teacher did not correspond exactly to the behaviour cluster in the learning platform. In addition, no significant differences were found in the level of satisfaction between the two kinds of teacher. The main contribution this study makes is to provide a detailed description of the training stage as well as the materials required for its repetition. Further analytical studies are required on teacher perception of training programmes in digital teaching in order to provide personalised training proposals that lead to an effective use of teaching in digital environments.

## Introduction

### Self-Regulation in Higher Education

Recent changes in higher education reinforce students’ active role in their learning and skills development. Students’ characteristics in terms of academic background, capacities and motivation are assumed as relevant variables in teaching planning; particularly in the case of first year students. Internationally, the literature points to high levels of underachievement and dropout rates for first year students (Bernardo et al., 2017; Páramo Fernández et al., 2017), which can be related to the fact that students commence their higher education studies with little knowledge and few skills in learning strategies or with little information about how to learn new curricular content (Kramarski and Michalsky, 2009).

If they are to ensure an autonomous and active role, students need appropriate levels of autonomy or self-regulation strategies in their learning. Zimmerman (2008) identifies three basic moments in learning self-regulation: planning, performance (monitoring) and self-evaluation. During these phases, an ensemble of thoughts, feelings and actions can be planned, implemented and adjusted by students to improve motivation, learning and achievement (Zimmerman, 2008; Zeynali et al., 2019). It is also important to regulate emotions (Pekrun et al., 2011) in order to achieve optimal performance.

Planning encompasses cognitive processes, prior knowledge, frequent habits and behaviours, as well as motivation and initial expectations. Two processes converge in this first phase: task analysis and demands, and expectations and self-efficacy perceptions (Boekaerts and Niemivirta, 2000). The main impact of good planning translates to an appropriate definition of goals and outlines the strategic plan required to achieve them (Zimmerman, 2013). Performance or execution monitoring is related to what occurs during learning; for example, levels of motivation, attention and self-monitoring (Schunk and Ertmer, 2000; Weinstein and Acee, 2018). These are clearly decisive processes in terms of learning quality and learning outcomes; in other words, with regard to the internal or external feedback that students can receive during task execution (Cervone, 1993; Schunk, 1995; Rheinberg et al., 2000; Kubik et al., 2021). Finally, self-evaluation occurs after task completion and after the achievement obtained has been analysed. Good self-regulation skills enable students to balance initial objectives and learning outcomes, to review the directions taken and the choices made, to consider contextual variables and to take into account all these variables in order to evaluate outcomes or performance and so produce self-evaluation, self-reinforcement and causal attributions (Bandura, 1986; Schunk, 1996; Zimmerman, 2000).

Self-regulation is a complex construct and authors recognise its multidimensionality. Instruments to evaluate self-regulation strategies or skills usually integrate the domain of basic knowledge, cognitive, metacognitive, emotional and motivational student resources (Zimmerman, 2000; Zimmerman and Schunk, 2011). In a contextual approach, self-regulation includes not only traditional cognitive and motivational factors but also regulation of emotions (Calkins and Williford, 2009; Raftery and Bizer, 2009; McClelland et al., 2010; Pekrun et al., 2011; Liew, 2012), the domain of specific knowledge and the level of use of electronic equipment and information. In addition, in terms of cognitive and metacognitive components, authors now pay greater attention to learning strategies and approaches, working memory, inhibitory control or thinking flexibility rather than to classical intelligence or IQ (Carlson, 2003; Rothbart et al., 2011; McClelland et al., 2014; Valadas et al., 2017).

Self-regulation strategies are no doubt related to other student characteristics but are also dependent upon teachers’ teaching and evaluation practices. Curricula plans in different degrees can also be an important moderating variable in student self-regulation development. Several programmes are usually introduced in an effort to promote these skills, particularly self-regulation. Institutions and teachers might need to implement diagnostic techniques to identify those skills which are most absent (cognitive, metacognitive, motivational and emotional), dealing with specific student subgroups.

### Advanced Learning Technologies and Self-Regulated Learning

The use of technology and educational data mining techniques (EDM) form part of the Advanced Learning Technologies (ALT) methodology. ALT is triggering a revolution in the field of cognitive psychology and learning, since it facilitates both the development and evaluation of the teaching-learning process. Much of today’s learning is carried out in virtual spaces. These environments aid self-regulated learning (SRL; Azevedo et al., 2011, 2015) through a range of different virtual reality resources and hypermedia, such as avatars and serious games (Kretschmer and Terharen, 2019; Sáiz-Manzanares et al., 2020). Van De Weijer et al. (2020) found that the use of gamification enhances students’ cognitive skills and boosts motivation (Nappo et al., 2020) in high duration interventions (24 weeks). The use of executive functions (control and self-regulation) is particularly important vis-à-vis acquiring new concepts or learning that involves a high degree of difficulty. These skills are directly related to establishing goals and to planning, and acquiring these skills is linked to achieving successful educational responses (Huizinga et al., 2018). Implementing metacognitive strategies can be enhanced through the use of serious games. Nevertheless, such interaction entails the need to have experts in learning psychology, in the development of virtual environments as well as experts in artificial intelligence, since analysing the results of platform learners will provide insights into and shed light on what the most appropriate type of game is for each user. As a result, gamification emerges as a help in the more efficient use of executive functions (attention, inhibition of distracting elements, planning and self-evaluation) as well as increased motivation. Specifically, the use of gamified learning strategies within virtual learning environments (Learning Management Systems -LMS-) enhances the quality of learning and engenders greater student motivation compared to conventional forms of learning (Pinnell, 2015). Moreover, the value of the effect within the differences found ranges between *d* = 0.45—*d* = 0.72, implying a medium-high effect (Taub et al., 2018). This appears to be because these activities help information to be processed in the working memory and in the long-term memory and prevent task execution from being abandoned (Lumsden et al., 2017).

Moreover, the joint use of LMS and ALT enables interactions to be recorded (Azevedo and Gašević, 2019; Hosain et al., 2019; Noroozi et al., 2019). The use thereof accounts for over 72% of variance in student learning outcomes (Sáiz-Manzanares et al., 2019a). One possible reason is that the use of ALT boosts SRL learning and the use of metacognitive strategies (planning, evaluation and design of task solving; Hull et al., 2015) as well as student motivation (Zimmerman, 2005), all of which enhances personalised learning (Enembreck and Barthès, 2005; Sáiz-Manzanares et al., 2019b; Martín-Antón et al., 2020), learner autonomy (Remesal et al., 2017; Zorrilla-Pantaleón et al., 2021) as well as self-awareness and self-reflection (Taub et al., 2017; Nurmi et al., 2020).

Nevertheless, research is required into the design of such environments, since the mere use of virtual platforms by no means ensures effective learning (Yamada and Hirakawa, 2016; Park and Jo, 2017; Sáiz-Manzanares et al., 2017). Carefully designed methodological aspects (objectives, conceptual and procedural content, assessment criteria) as well as technological aspects (Sáiz-Manzanares et al., 2019a) must be applied if these environments are to foster the development of metacognitive strategies and self-regulation. Moreover, virtual learning platforms must embrace student follow-up systems so that teachers can track the behaviour of each of their students throughout the learning process (Jommanop and Mekruksavanich, 2019; Troussas et al., 2021; Krouska et al., 2021b; Sáiz-Manzanares et al., 2021b).

### Teacher Training in Higher Education in Effective Teaching in Virtual Environments

As has become clear through the previous points, the teaching-learning process in LMS, particularly in higher education contexts, involves addressing digital transformation. This has been hastened by the current situation triggered by the SARS-CoV2 pandemic (García-Peñalvo, 2021; Sáiz-Manzanares et al., 2021a). Said crisis is having an impact on the teaching-learning process, particularly in higher education, since it is leading to a situation of uncertainty which is reflected in emotional behaviour related to anxiety during the process, both amongst teachers and students alike (de la Fuente et al., 2021a). This prompts the need to develop teaching models based on preventing the situations of uncertainty that trigger anxiety (de la Fuente et al., 2021b). In order to meet the challenge of a true digital transformation in higher education, technological resources together with innovation in teaching processes must be introduced (García-Peñalvo and Corell, 2020). All of this leads to teacher training, which will need to focus on content handling of LMS and ALT resources (e.g., avatars, gamification and automatic feedback procedures). This challenge in terms of training is one of the goals of government authorities included in objective 5, quality of teaching, of the 2030 Agenda (Redecker and Punie, 2017; Jarillo et al., 2019). In this line, the European Commission has established a Framework for the Digital Competence of Educators (DigCompEdu; Redecker and Punie, 2017). DigCompEdu defines six levels of teaching staff competence: (A1) Newcomers (teachers who have had very little contact with digital tools); (A2) Explorers (teachers who have begun to use digital tools, but who lack a global or consistent approach, such that they need to expand their skills); (B1) Integrators (use and experiment with digital tools for a variety of purposes, seeking to determine which digital strategies function best in each context); (B2) Experts (use a range of digital tools with confidence, creativity and a critical eye to improve their professional activities. They are constantly expanding their repertoire of practical work); (C1) Leaders (use a wide range of flexible, comprehensive and effective digital strategies. They are a source of inspiration for other teachers); (C2) Pioneers (question the suitability of the contemporary digital and pedagogical practices which they themselves are experts in. They lead the way in innovation and are a model for younger teachers). The ultimate goal is to train professionals with skills in educational digitalisation in order to increase motivation and help students achieve efficient learning (Carbonero et al., 2017).

Moreover, training in digital skills amongst teachers, particularly within the framework of higher education, is a challenge that requires implementing formal training programmes (García-Peñalvo, 2021). The content of these training proposals in e-Learning or b-Learning spaces during the COVID-19 pandemic in higher education must take into account (Collazos et al., 2021; de la Fuente et al., 2021b):

– Frequent interaction through technological resources at specific times (scheduled synchronised sessions).
– Expectations of normality in work during the teaching-learning process.
– Fostering collaborative work and assessment systems with feedback on the process.
– Facilitating SRL through technological resources in LMS.
– Incorporating personalised consultation (through videoconferences, forums or chats).
– Safeguarding students’ emotional state, avoiding loneliness in the net.

In addition, gaining an insight into teachers’ perception of the educational processes related to the use of technological resources in teaching as well as distinguishing between inexperienced and experienced teachers is key to improving teaching processes in today’s society, particularly given the current worldwide pandemic (Krouska et al., 2020). Moreover, it is important to develop teaching models that take into account the emotional and social aspects of cognitive and metacognitive development within the framework of e-learning or b-learning, which is undoubtedly here to stay (Dumulescu et al., 2021). Furthermore, designing these learning environments is key to the success of the teaching-learning process (Collazos et al., in press).

Taking into account the conclusions to emerge from the previously mentioned studies, the research questions (RQ) for the study were:

1. “Will the behaviour patterns of university teachers during a training activity in digitalisation in Moodle depend on whether they are inexperienced or experienced teachers?”
2. “Will behaviour clusters in LMS correspond to the differentiation between the type of teacher (inexperienced or experienced)?”
3. “Will the level of satisfaction with the training activity in digital teaching depend on the type of teacher (inexperienced or experienced)?”

This study applied mixed methods, merging quantitative and qualitative analyses (Anguera, 1986; Castañer-Balcells et al., 2013). Specifically, a quantitative and qualitative study was used to test RQ1 and RQ2, and a quantitative study was carried out to test RQ2.

## Materials and Methods

### Participants

We worked with a total sample of 23 teachers, 15 experienced teachers (with over 15 years teaching in higher education), nine females and six males, and 8 inexperienced teachers (with 1–2 years teaching experience in higher education), seven female and one male, from four universities (University of Burgos, University of Oviedo, University of Minho and University of Valladolid). Experienced teachers were aged between 45 and 60, and inexperienced teachers were aged between 25 and 30. Prior to commencing the study, all the participants were informed of the aim of the research and their written consent was requested. A convenience sample was used to select participants. Participants were selected by each partner involved in the SmartArt project, following the guidelines set out in the project report a learning activity is organised for two students and two teachers for each partner (eight students and eight teachers in all) chosen at random from amongst the participating organisations. However, the number of participants was increased depending on the requests put forward by each partner. Throughout the study, 2 experimental deaths were detected in the group of experienced teachers.

### Instruments

#### Initial Survey on Prior Knowledge of ALT

An *ad hoc* survey was drawn up to ascertain participants’ level of prior knowledge of the training activity related to their know-how and application of teaching resources in virtual learning environments (Sáiz-Manzanares, 2021). The survey consisted of nine closed response questions, measured on a 1–5 Likert-type scale, with 1 being the lowest level of prior knowledge and 5 the highest. Survey reliability was determined by applying the composite reliability index, Omega index, with the value for the general scale being *Ω* = 0.90. Two open response questions were also included (1. What are your expectations towards the training activity? What would you like to learn in the training activity?). This survey is available in Supplementary Table S1.

#### Application Web UBUMonitor

UBUMonitor is an open-code and free computer application (Ji et al., 2018). The application runs in the client and is implemented through Java, and it has a graphic interphase developed in JavaFX. The application is connected with the chosen Moodle server through web services and the API REST provided by the server. When no web services are available to retrieve specific data, web scraping techniques are also used. All the communication between the Moodle server and the client UBUMonitor is encrypted *via* HTTPS for security reasons. As a result of these queries, data are obtained in JSON and CSV format, processed and transformed into Java objects in the client. Java and webpages are applied with different graphic libraries of JavaScript within the desktop application in order to visualise the data gathered. The application includes six modules: visualisation (which offers frequency representation in different graphics: Heat Map, Boxplot, Violin, Scatter, etc.), comparisons, forums, dropout rate risk (locating students who have failed to log on for 7–15 days at certain moments of the course), Calendar of events and Clustering (finding clusters by applying different algorithms such as *k*-means ++, Fuzzy *k*-means, etc.). Specifically, in this study we used the visualisation module, which allows for an analysis of access frequency in components, events, sections or courses seen in Moodle, with options to analyse the registers in different graphics. In this work, we opted for the Heat Map visualisation technique, since it provides the results with numerical and colour intensity visualisation throughout the course during the training activity. The use of visualisation techniques such as Heat Map is felt to be very useful to assess user behaviour in LMS (Dobashi et al., 2019). The UBUMonitor application may be downloaded free at https://github.com/yjx0003/UBUMonitor.

#### Training Programme for University Teachers

**Figure 1:**
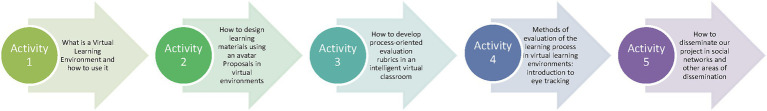
Structure of the training in synchronised phases.

This programme was implemented in the LMS based on Moodle UBUVirtual. It was also based on the use of ALT, grounded in the use of gamification in self-assessment systems to promote SRL. The training programme lasted 4 weeks and consisted of a synchronised online phase made up of five 3-h training sessions. These sessions were carried out in UBUVirtual through the joint communication and collaboration Teams platform. A description of the phases of the synchronised sessions can be seen in Figure 1. The documents related to the teaching staff training sessions may be consulted at: https://bit.ly/3vsS94l.

**Figure 2:**
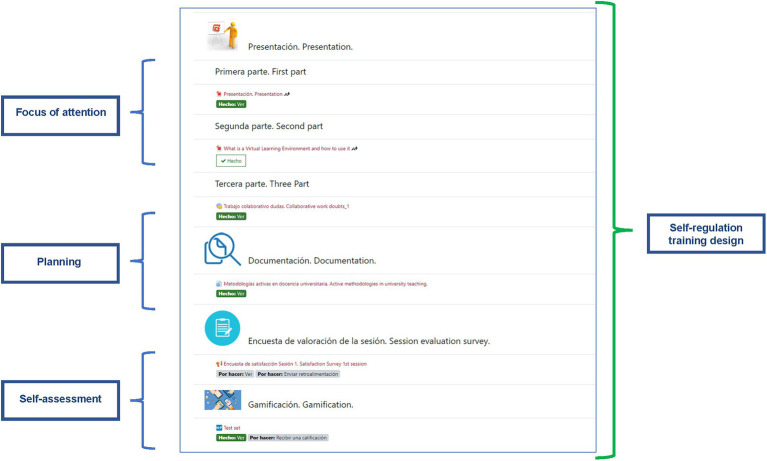
Didactic structure of each of the training sessions.

Each of the sessions had a consistent pedagogical structure comprising presentations on the topics dealt with during each session: a collaborative work chat to deal with doubts, complementary documentation, gamification activities to understand the concepts of the topic and a satisfaction survey for the training activity. Figure 2 sets out the structure. Training in all the sessions was offered in Spanish and in English. The specific content of the synchronised training sessions may be consulted in Supplementary Table S2. This training structure follows the approach of acquiring executive control strategies, since these initially seek to focus participant attention on the content to be dealt with in each unit. They then direct planning strategies in order to establish the learning goals related to the content. Finally, they focus on the acquisition of self-evaluation strategies, in this case through gamified learning techniques with automatic feedback and with satisfaction surveys that encourage reflection on the learning process.

The gamification activities designed for each training session can be seen in Supplementary Table S3. All of them were designed using the HTML5 package (H5P). H5P is a totally free and open technology, with an MIT licence. Information may be found at https://h5p.org/. H5P is a resource that may be implemented in LMS similar to Moodle, WordPress or Drupal and which enables educators to create different types of content. The following resources were specifically used in this study: Drag the Words (allowing challenges to be created based on text in which users have to drag words into gaps in the sentences), Find the words (users have to find a series of keywords in the grid) and Multiple Choice (multiple choice questions). It also includes instant feedback on the correct options and the reasons for these and True/False Question (refers to true-false questions). All of these serious games involved feedback on the answers as well as information on progress.

The training programme also involved a synchronised training phase that took place over a three-week period. During this phase, teachers had to develop a teaching design proposal for each university group to be applied in a virtual learning environment. This design had to include one of the tools seen during the synchronised training phase. Interaction was by email or through a forum set up for the purpose on the UBUVirtual platform. This training design was similar in structure to the one which teachers would be expected to include during their teaching in higher education.

#### Satisfaction Survey With the Synchronised Sessions

An *ad hoc* survey was designed to gauge participant teacher satisfaction with the synchronised training activity. The survey was made up of four closed response questions measured on a 1 to 5 Likert-type scale, where 1 reflects the lowest level of satisfaction and 5 the highest, in which satisfaction is assessed with the concepts, materials, complementary information and work time devoted to the activity, together with three open questions [(1) indicate which aspects need to be extended in this part of the course, (2) indicate the aspects to be removed from this part of the course, and (3) suggestions for improvement]. Survey reliability was attained by applying the composite reliability index, Omega, and which gave **Ω** = 0.62. This instrument is available in Supplementary Table S4.

#### Satisfaction Survey With the Training Activity

This survey was designed *ad hoc* and was based on the assessment criteria of the European Commission for the Evaluation of Learning Activities in European projects. The survey is made up of 14 closed response questions, measured on a 1–5 Likert-type scale, where 1 reflects the lowest level of satisfaction and 5 the highest. Survey reliability was attained by applying the composite reliability index, which gave Omega, **Ω** = 0.96.

The survey also included two open response questions [(1) which of the gamification materials have you found most useful for understanding the concepts? and (2) what elements would you introduce or increase in gamification materials?]. This instrument is available in Supplementary Table S5.

### Procedure

This research was carried out as part of the “Self-Regulated Learning in SmartArt (SmartArt)” project funded by the European Commission. The aims of the project focus on designing SRL-based virtual intelligent classrooms and the use of avatars to facilitate personalised and independent student learning. For further information, see https://srlsmartart.eu/en.

The project was backed by a favourable report issued by the University of Burgos Bioethical Committee, No. IR 27/2019, the coordinating university. The project was to contain a training phase aimed at university teachers from partner universities and which dealt with teaching strategies in virtual learning platforms based on self-regulated learning through the use of technological resources.

**Figure 3:**
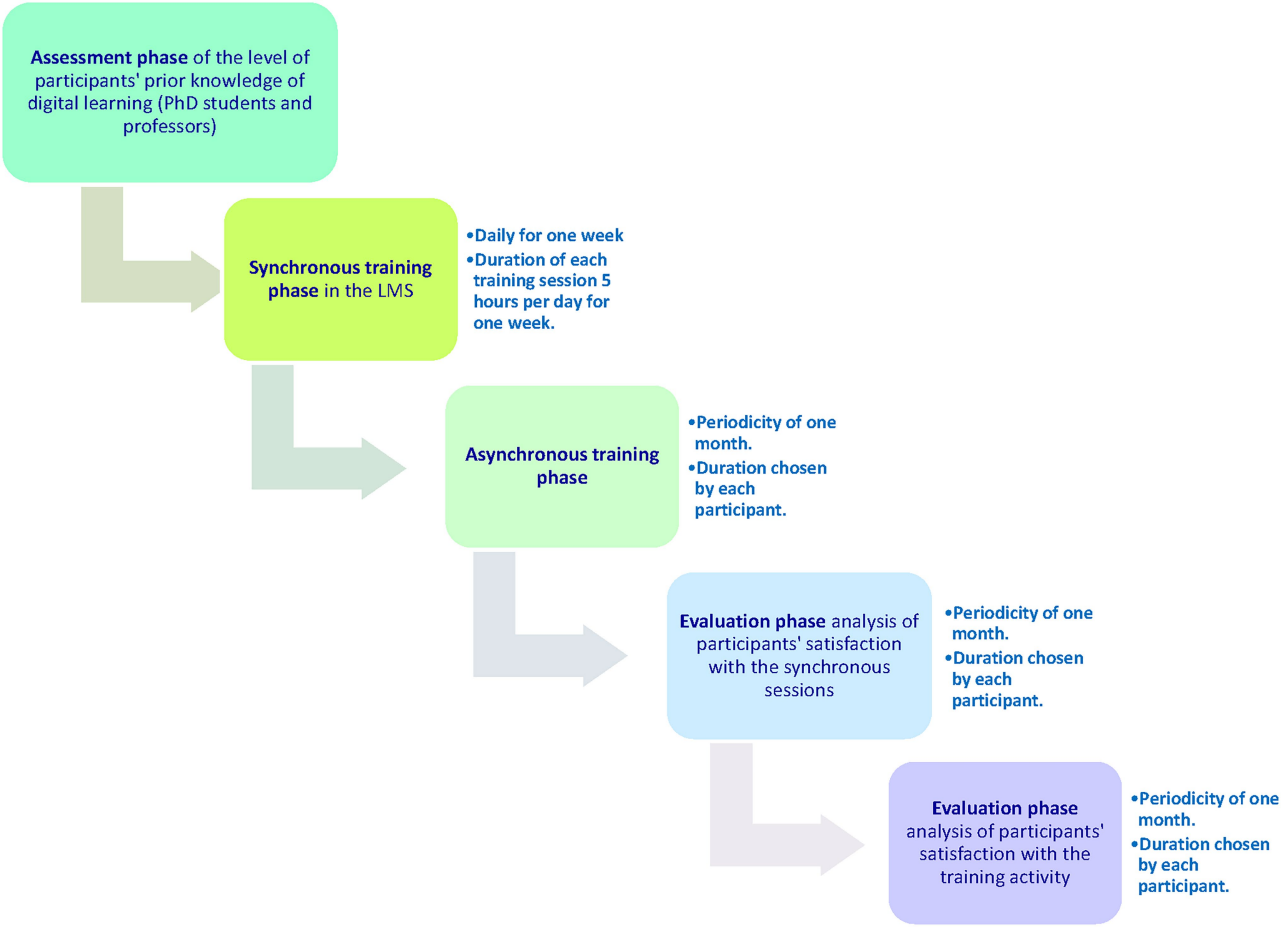
Diagram of the procedure used in this study.

Prior to commencing the study, participating teachers’ level of prior knowledge in digital teaching was evaluated. To this end, an *ad hoc* survey was designed—see instruments section. The online training stage, consisting of a synchronised phase (lasting a week), was then carried out. After each synchronised training session, a satisfaction survey was conducted with the synchronised sessions (see “Instruments” section). There was also a non-synchronised phase (lasting 3 weeks). Finally, once the training activity had concluded, participants were given an *ad hoc* satisfaction survey on the activity (see instruments section). A diagram of the procedure used in this study can be seen in Figure 3.

### Data Analysis

#### Prior Analysis

Before testing the RQ, a normality study was carried out on the sample, for which asymmetry and kurtosis analyses were applied. The SPSS v.24 statistical package (IBM, 2016) was used for this purpose.

#### Hypotheses Testing

In order to test the RQ, quantitative and qualitative studies were performed. With regard to the latter, a descriptive design was applied (Campbell and Stanley, 2005), and a comparative longitudinal design was used for the latter (Flick, 2014).

As regards the quantitative study, since some of the asymmetry indicators did not ensure normal distribution and the *n* of subjects in the sample was below 30, a non-parametric statistic was applied. Specifically, to test RQ1 and RQ3 the Mann–Whitney *U* test for independent samples was used (Mann and Whitney, 1947), for which the SPSS v.24 statistical package was used (IBM 2016; see Equation (1)).


Ui=n1+n2+ni(ni+1)/2−Ri

where n_1_ will be equal to the n of group 1, and *n*_2_ will be equal to the n of group 2, and *R*_i_ is construed based on the sum of the ranges of one of the samples chosen at random. The value of the effect size was determined by applying the formula of eta squared [*η*^2^; see Equation (2)]. As regards the interpretation of the values, and following Cohen (1988), a very small effect size was considered to be one between 0 and 20, small between 20 and 49, medium between 50 and 69, with over 70 being considered as high.


$$\eta^{2}=\frac{\mathbb{Z}}{N}-1$$

With regard to testing RQ2, cluster analysis was used, applying the *k*-Means ++ algorithm. This algorithm is applied to select the initial values of the centroids for the *k*-means clustering algorithm. This was proposed in 2007 by Arthur and Vassilvitskii (2007) as an approximation algorithm to address the NP-hard *k*–means problem: in other words, as a way of avoiding the occasionally poor clustering found by the standard *k*-means algorithm [see Equation (3)].


$$D^{2}\left(\mu_{0}\right)\leq 2D^{2}\left(\mu_{i}\right)+2{\left|\left|\mu_{i}-\mu_{0}\right|\right|}^{2}$$

being μ_0_ the initial point selected and D the distance between point *μ_i_* and the centre closest to the cluster. Having chosen the centroids, the process is like the classical *k*-means. To find this, the UBUMonitor tool was used (Ji et al., 2018).

Also used was Pearson’s contingency coefficient C (which expresses the intensity of the relation between two or more qualitative variables, and which is based on comparing the sequences of two characteristics with the expected frequencies). This is calculated by calculating *χ* 2 , adding the categorisations of the two judges in the analysis of subjects’ responses in all the analysis units and then removing empty categories (López-Roldán and Fachelli, 2015; see Equation (4)). The statistical package SPSSv.24 (IBM, 2016) was used to determine this.


C=(χ2N+χ2)

As regards the qualitative study, Heat Map visualisation techniques derived using the UBUMonitor tool (Ji et al., 2018) were used in RQ1, and in RQ3 frequency analysis was used on the categorisation criteria for the open answers to the initial and final evaluation surveys carried out using ATLAS.TI 9 software (Atlas.ti, 2020).

## Results

### Prior Analysis

Prior to commencing the study, a check was carried out on the distribution of the sample vis-à-vis their previous knowledge in digital teaching. Asymmetry values were adjusted in all the items except in items 4 and 9, in which a slightly higher value was seen (values over |2.00| are considered extreme). As regards the kurtosis values, no extreme values were found (values between |8.00| and |20.00| are considered acceptable; Bandalos and Finney, 2001), see Table 1. As a result, a normal distribution was not considered, and a non-parametric statistic was applied to test the research questions.

**Table 1:** Descriptive statistics and asymmetry and kurtosis values in the initial survey on prior knowledge.

Question	*M(SD)*	*Asymmetry*	*ESA*	*Kurtosis*	*ESC*
I believe that the teaching-learning process should be interactive between teacher and student.	5.00(0.00)	-	0.56	–	1.91
I have a knowledge of how to design virtual learning platforms.	2.81(1.18)	0.16	0.56	−1.17	1.91
I have a knowledge of how to design process-oriented feedback.	3.00(1.17)	0.00	0.56	−0.47	1.91
The feedback provided by the teacher on the student’s practice should be clear, positive and task-dependent.	4.88(0.33)	−2.51	0.56	4.90	1.91
I have a knowledge of eye tracking methodology.	2.44(1.12)	−0.13	0.56	−1.46	1.91
I have a knowledge of how to design learning-oriented gamification activities.	3.25(1.30)	−0.33	0.56	−0.99	1.91
I have a knowledge of project dissemination in social networks.	2.81(1.07)	0.08	0.56	−0.27	1.91
I have previously used gamification experiences as a learning resource.	3.06(1.52)	0.12	0.56	−1.56	1.91
I have previously used the Alexa skill to monitor learning activities.	1.44(1.00)	2.28	0.56	3.95	1.91

ESA = Standard Error Skewness.ESC = Standard Error Kurtosis.

In the qualitative study of the open response questions, the questions were first categorised and then analysed with the Atlas.ti 9 qualitative analysis program, applying percentage analysis to the categorised responses. Results indicate there were two kinds of interests amongst participating teachers; one part preferred to learn about basic resources for implementing teaching in virtual spaces (33.33%), and another group requested advanced techniques (29.0%). A general interest was also noted in specifically learning about SRL techniques through avatars and gamification techniques (28.57%).

#### Testing the Research Questions

In order to test RQ1 “Will the behaviour patterns of university teachers during a training activity in digitalisation in Moodle depend on whether they are inexperienced or experienced teachers?”

#### Quantitative Study

An analysis was first carried out to ascertain whether there were significant differences in interaction in the training platform between inexperienced or experienced teachers. In order to test this, we applied the non-parametric Mann–Whitney *U* test of differences between independent samples (see Table 2). Two experienced teachers who signed up for the activity later did not take part for personal reasons.

**Table 2:** Descriptive statistics and Mann–Whitney *U* test comparing teachers (inexperienced or experienced) on the UBUVirtual training platform during the synchronised training phase.

Synchronised training sessions	Session content	Group 1 Inexperienced *n* = 8	Group 2 Experienced *n* = 13	Mann–Whitney *U*	*p*	*Z*	*η^2^*
		*M*(*SD*)	*M*(*SD*)				
Session 1	Definition and use of Virtual Learning Environments (VLE)	51.75(47.51)	24.76(40.09)	35.00	0.21	−1.25	0.08
Session 2	Design of materials and use of avatars	37.00(43.38)	21.54(43.19)	38.00	0.31	−1.02	0.05
Session 3	Design of rubrics in the VLE	36.00(38.29)	4.00(9.55)	9.00	0.001^*^	−3.24	0.52
Session 4	Use of Learning Analytics Systems in the VLE	43.25(25.79)	8.54(16.58)	10.50	0.002^*^	−3.10	0.48
Session 5	Dissemination in social networks	36.00(45.46)	15.85(17.70)	32.00	0.144	−2,32	0.27

* *p < 0.05*.

*Z = Z de Kolmogorov–Smirnov; effect size η^2^*. M = Mean. SD = Standard Deviation.

Significant differences were found in platform interaction between inexperienced teachers and experienced teachers in session 3 (designing rubrics in VLE) and a medium effect value (*η*^2^ = 0.50), session 4 (use of Learning Analytics Systems in VLE) and small effect value (*η*^2^ = 0.46), in favour of the group of inexperienced teachers and a small effect size (*η*^2^ = 0.46).

#### Qualitative Study

**Figure 4:**
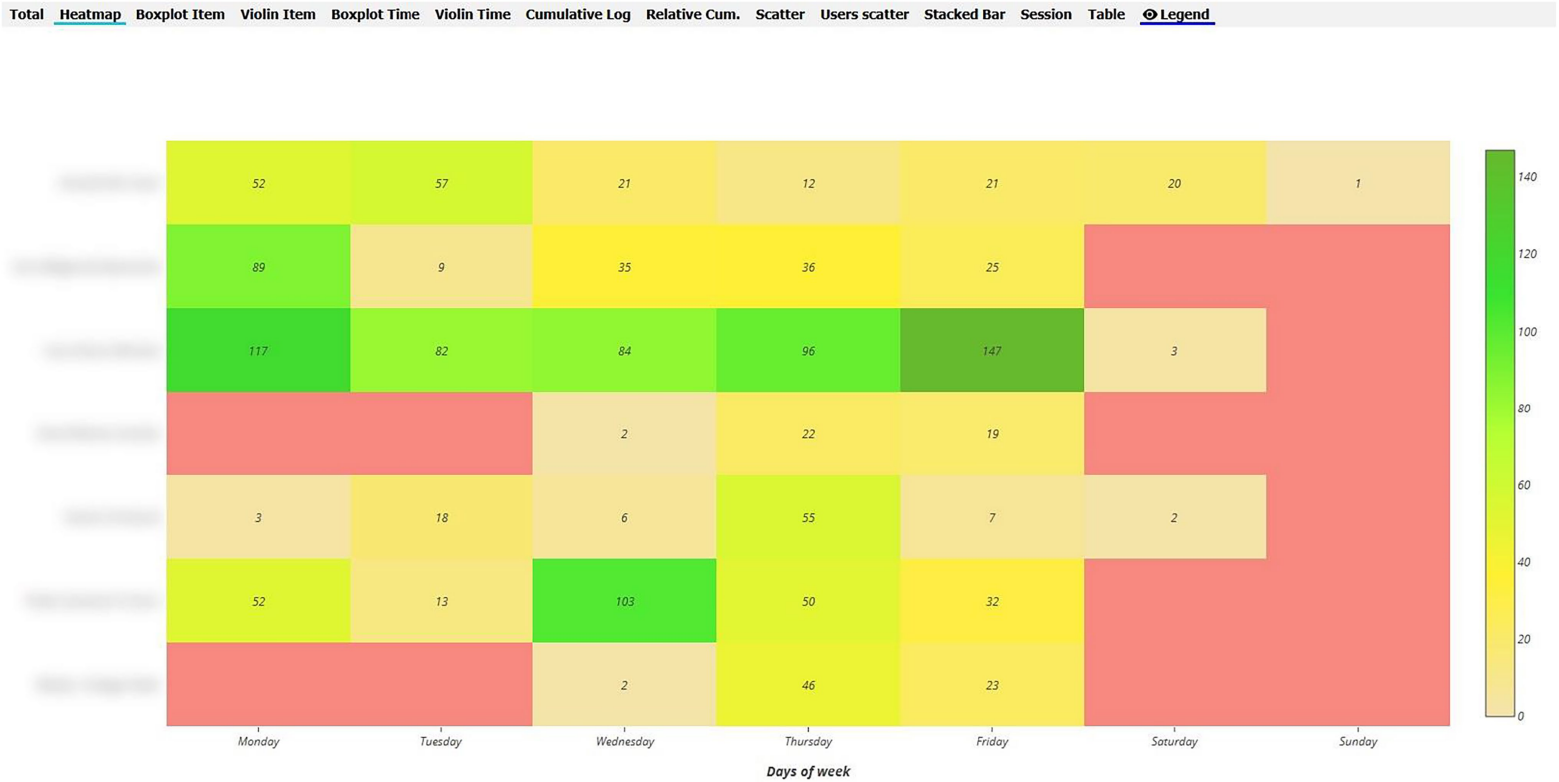
Heat Map of inexperienced teacher behaviour in the Moodle platform during the synchronised phase.

**Figure 5:**
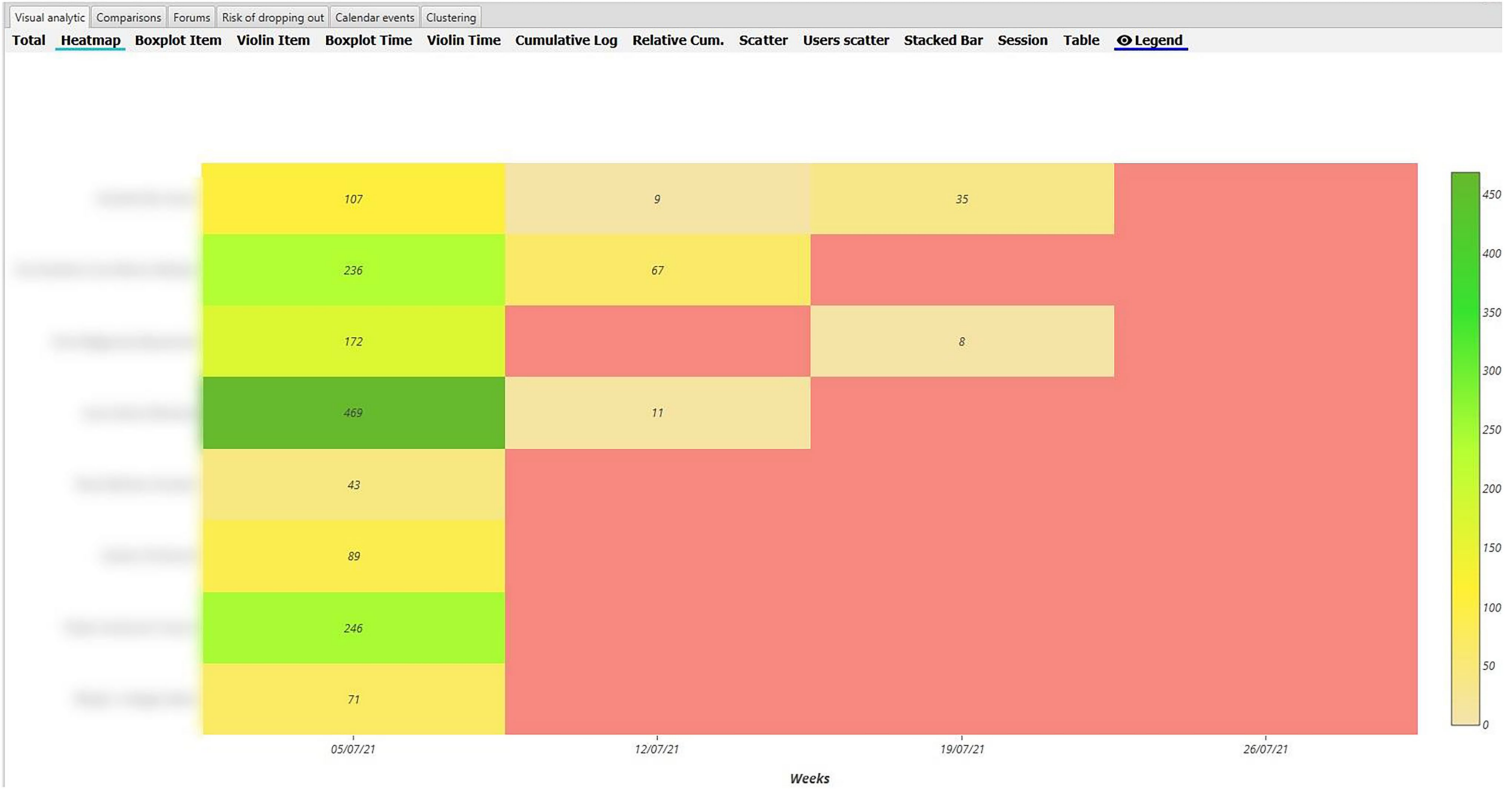
Heat Map of inexperienced teacher behaviour in the Moodle platform during the non-synchronised phase.

In order to analyse RQ1, the heat maps in the various Moodle components were pinpointed, distinguishing between the maps of inexperienced vs. experienced teachers during the synchronised and non-synchronised interaction phases. With regard to behaviour analysis, greater interaction was evident in the UBUVirtual platform during the synchronised phase when compared to the non-synchronised phase for both types of teacher, although interaction frequency was more intense amongst inexperienced teachers (see Figures 4, 5).

**Figure 6:**
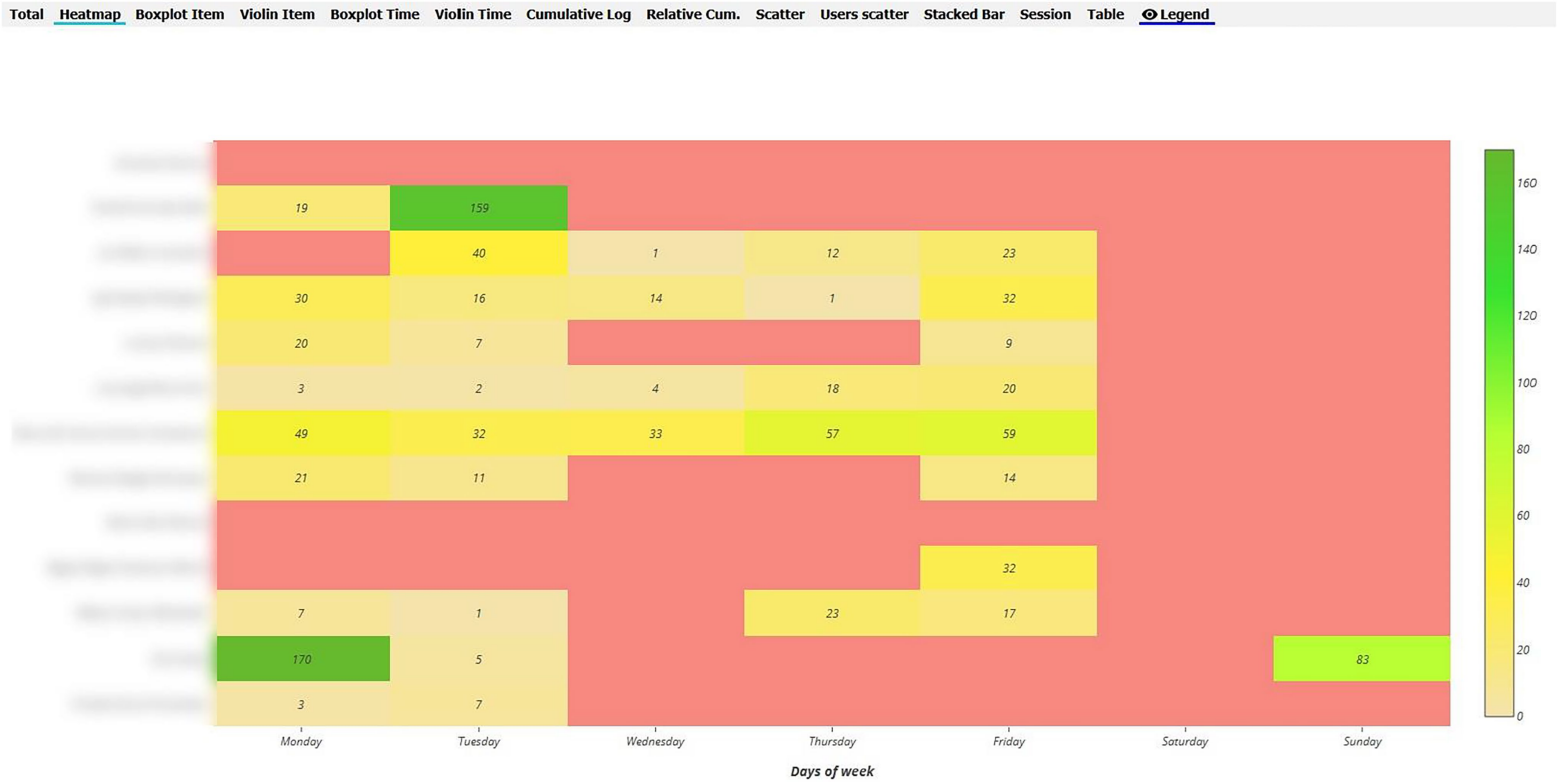
Heat Map of experienced teacher behaviour in the Moodle platform during the synchronised phase.

**Figure 7:**
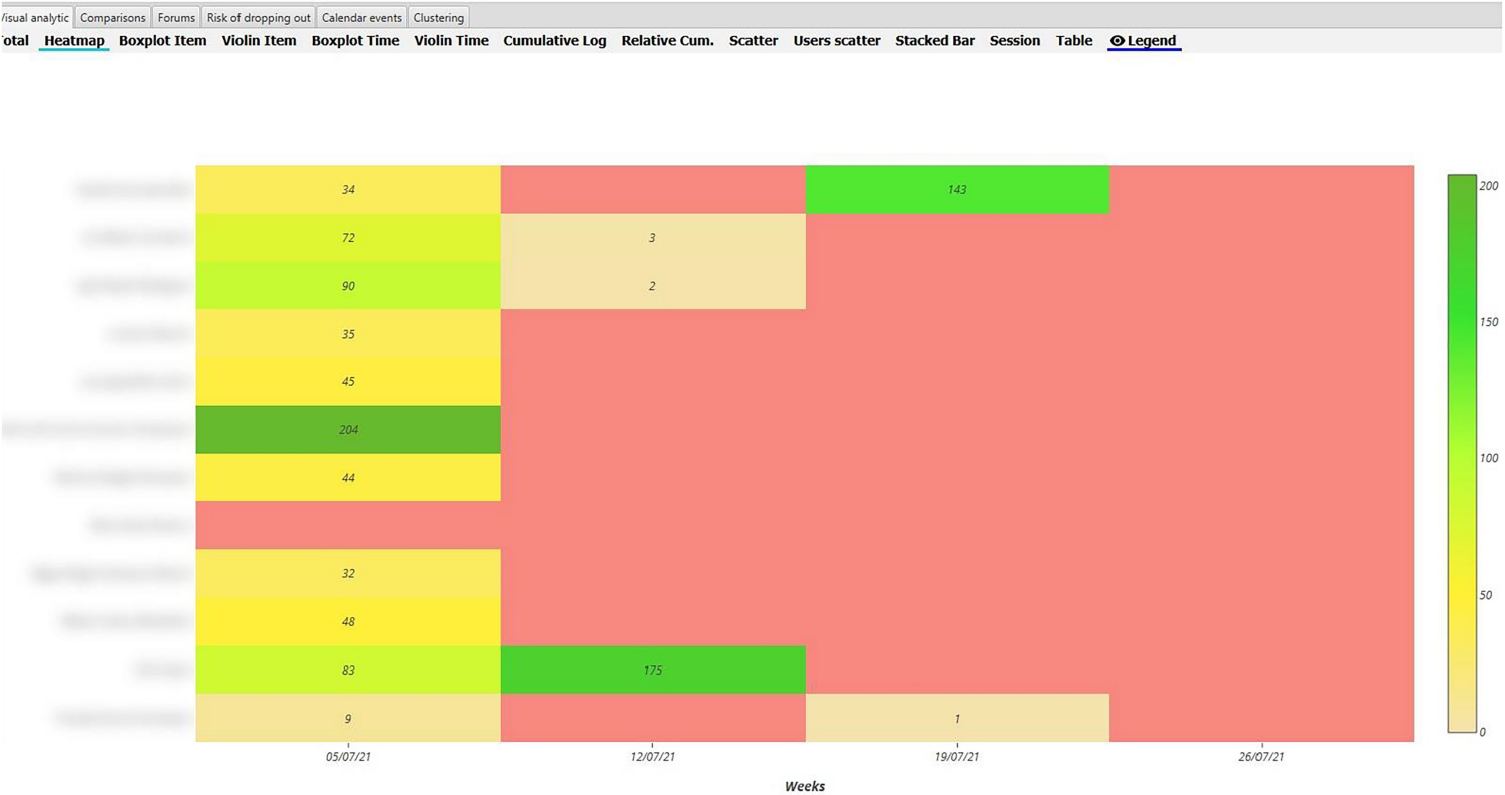
Heat Map of experienced teacher behaviour in the Moodle platform during the non-synchronised phase.

As regards the analysis of behaviour during the non-synchronised training phase, a decrease was seen in interaction frequency in both types of teacher (inexperienced and experienced), although interaction frequency was greater amongst inexperienced teachers (see Figures 6, 7).

**Figure 8:**
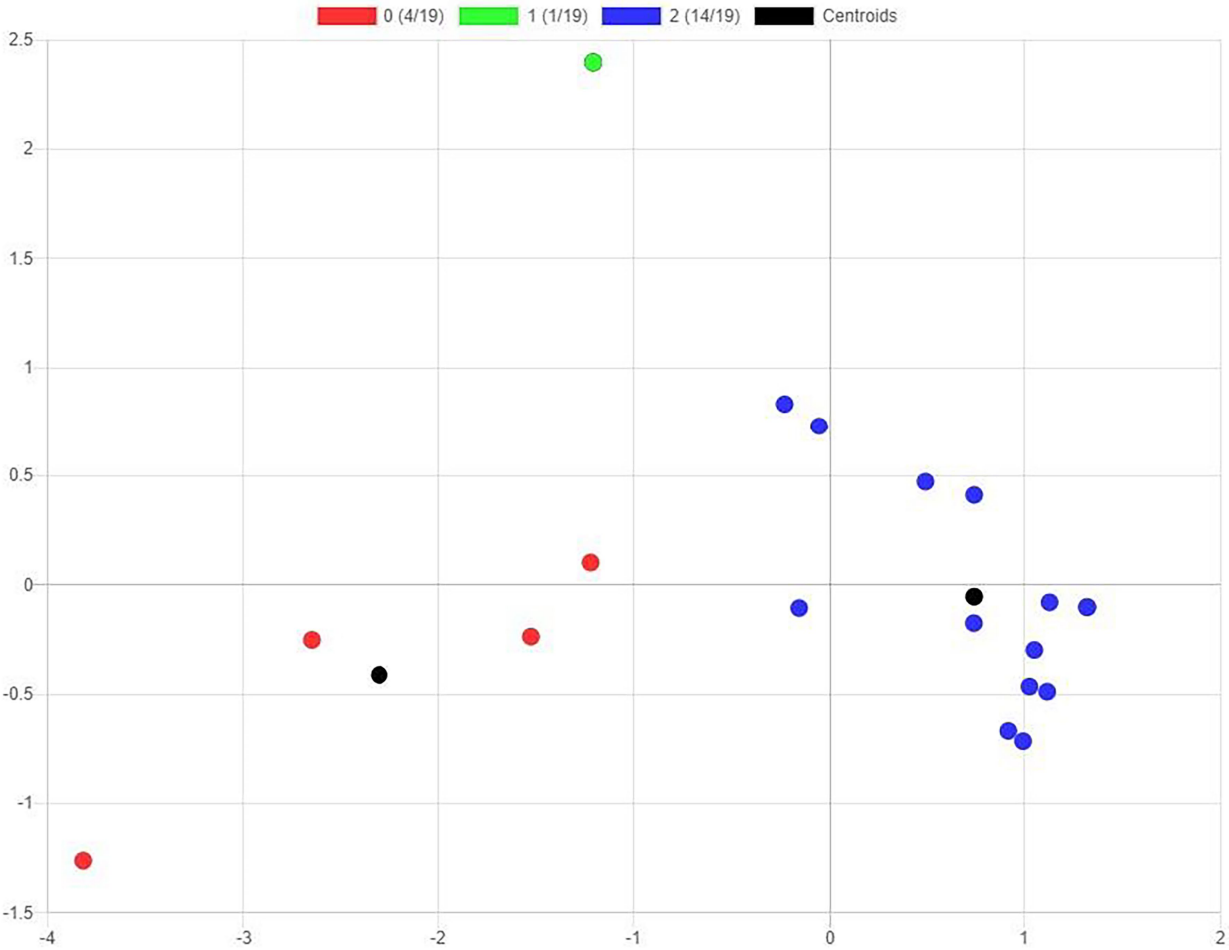
Cluster analysis with the *k*-means ++ algorithm.

In order to test RQ2 “will behaviour clusters in LMS correspond to the differentiation between the type of teacher (inexperienced or experienced)?,” we first used an eight-cluster analysis with regard to the number of registers in the platform of the completed activity, for which the *k*-means ++ algorithm was applied (see Figure 8).

We then designed a cross-reference table between the allocation cluster and inclusion in the group of inexperienced or experienced teachers (see Table 3). We also found the coefficient of contingency, which obtained a value of *C* = 0.41 to be non-significant *p* = 0.28. This indicates there is no strong correspondence between the cluster allocated and the type of group to which the teacher belongs. Non-significance might be due to the small number of elements in the sample.

**Table 3:** Cross-reference table between the values of the cluster allocation and the type of teacher; inexperienced vs. experienced.

Type of teacher	Cluster			Total
	0	1	2	
Inexperienced	5	2	1	8
Experienced	10	3	0	13
Total	15	5	1	21

In order to test RQ3 “Will the level of satisfaction with the training activity in digital teaching depend on the type of teacher (inexperienced or experienced)?” we applied the non-parametric Mann–Whitney U test of differences between independent samples.

An analysis of satisfaction with the overall training activity was performed.

No significant differences were found between the group of inexperienced teachers vs. experienced teachers in the level of satisfaction in any of the items contained in the satisfaction survey for the training activity. The effect value was seen to be low in all the items. Moreover, mean satisfaction scores were high in all the items, with the means interval ranging from 4.33 to 5 out of 5 (see Table 4). The significance of the coefficients may be explained by the sample size, which in this study was small.

**Table 4:** Descriptive statistics and Mann–Whitney *U* test for the results in the satisfaction survey for the training activity in participating teachers (inexperienced vs. experienced).

Final evaluation survey on the activity	Group 1 Inexperienced *n* = 8	Group 2 Experienced *n* = 13	*U* Mann–Whitney	*p*	*Z*	*η^2^*
	*M*(*SD*)	*M*(*SD*)				
Communication with the meeting coordinator.	5(0.00)	5(0.00)	24	0.65	0	0.00
Learning activity agenda.	4.83(0.41)	4.63(0.74)	21.50	0.65	−0.45	0.01
Presentation on the ongoing progress by the project coordinator.	4.67(0.52)	4.63(0.74)	23.00	0.87	−0.16	0.00
Time management.	4.50(0.55)	4.63(0.74)	19.50	0.49	−0.69	0.02
Atmosphere and communication among attendees.	4.50(1.23)	4.38(1.19)	22.50	0.79	−0.27	0.00
Would you be interested in using the tools proposed in your job?	4.67(0.82)	4.38(1.10)	19.50	0.47	−0.73	0.03
Do you consider that the tools presented are easy to use when teaching?	4.33(1.21)	3.63(1.30)	14.50	0.20	−1.29	0.08
Do you consider that specific training is necessary to use the tools presented?	4.50(0.84)	4.38(0.74)	18.00	0.41	−0.83	0.03
Would you like to spread the proposed tools among your colleagues?	4.50(0.84)	4.38(0.74)	21.00	0.66	−0.44	0.01
Quality of the virtual environment in which the training action was carried out.	4.67(0.52)	4.63(0.74)	23.00	0.87	−0.16	0.00
The gamification activities have made it easier for me to understand the concepts.	4.00(0.89)	4.25(1.04)	19.00	0.49	−0,69	0.02
Their satisfaction with the duration of the training activity is.	4.50(0.84)	4.50(0.76)	23.50	0.94	−0.78	0.03

*Z = Z de Kolmogorov–Smirnov; effect size η^2^*. M = Mean. SD = Standard Deviation.

As regards the analysis of the responses to the open questions, the latter were categorised and analysed with the Atlas.ti 9 qualitative analysis program, applying percentage analysis of the categorised responses. It was found that the training activities that aroused the greatest interest amongst teachers were those related to working with avatars to provide SRL (44.44%) and designing gamification activities to provide student self-evaluation (22.22%). In addition, 73% of teachers considered that the training activity fitted in well with the time and content, with 12.5% indicating that they would reduce slightly the time devoted to the activities during the synchronised phase.

An analysis of satisfaction with each of the synchronised training sessions was also carried out. To do this, the Mann Whitney *U* test was applied to the responses of the satisfaction survey conducted for each synchronised session. The mean satisfaction scores in the evaluation of all the synchronised training sessions were high, since they ranged from 4.06 to 4.82 out of 5 in the group of inexperienced teachers and from 3.79 to 4.80 out of 5 in the group of experienced teachers. Nevertheless, significant differences did emerge in the satisfaction with training session 2 (design of materials and use of avatars), session 4 (use of Learning Analytics Systems in VLE) and session 5 (dissemination in social networks) with regard to the clarity of the concepts explained, in favour of the group of inexperienced teachers (see Table 5). In all cases, the effect value was low.

**Table 5:** Descriptive statistics and Mann–Whitney *U* test for the results in the satisfaction survey of the training activity in teachers participating in the satisfaction surveys for the synchronised sessions.

Final evaluation survey for the activity	Group 1 Inexperienced *n* = 8	Group 2 Experienced *n* = 13	*U* Mann–Whitney	*p*	*Z*	*η^2^*
	*M*(*SD*)	*M*(*SD*)				
Training session 1. Definition and use of VLE						
The concepts dealt with in this section were clear to me.	4.67(0.47)	4.17(0.60)	29.00	0.08	−1.74	0.15
The materials presented in this session have proven useful for my teaching.	4.15(0.83)	4.25(0.54)	47.50	0.74	−0.34	0.01
The complementary information has proven to be useful to me.	4.42(0.50)	4.32(0.51)	51.50	0.97	−0.04	0.00
This session requires more work time.	3.59(1.50)	3.79(0.64)	51.50	0.97	−0.04	0.00
Training session 2. Design of materials and use of avatars						
The concepts dealt with in this section were clear to me.	4.82(0.37)	4.37(0.33)	18.00	0.009^**^	−2.62	0.34
The materials presented in this session have proven useful for my teaching.	4.68(0.71)	4.33(0.65)	27.50	0.06	−1.91	0.18
The complementary information has proven to be useful to me.	4.55(0.73)	4.28(0.50)	33.50	0.16	−1.41	0.10
This session requires more work time.	4.12(0.99)	3.81(0.68)	44.00	0.55	−0.60	0.02
Training session 3. Design of rubrics in VLE						
The concepts dealt with in this section were clear to me.	4.44(0.73)	4.54(0.32)	50.00	0.88	−0.15	0.00
The materials presented in this session have proven useful for my teaching.	4.32(1.17)	4.74(0.21)	47.50	0.72	−0.36	0.01
The complementary information has proven to be useful to me.	4.46(0.91)	4.80(0.17)	47.50	0.72	−0.36	0.01
This session requires more work time.	4.25(1.04)	3.85(0.99)	35.50	0.18	−1.34	0.09
Training session 4. Use of Learning Analytics Systems in VLE						
The concepts dealt with in this section were clear to me.	4.47(1.04)	4.30(0.27)	25.50	0.04^*^	−2.07	0.21
The materials presented in this session have proven useful for my teaching.	4.66(0.69)	4.62(0.32)	34.50	0.17	−1.39	0.10
The complementary information has proven to be useful to me.	4.66(0.69)	4.62(0.32)	34.50	0.17	−1.39	0.10
This session requires more work time.	4.42(0.49)	4.62(0.32)	48.00	0.76	−0.31	0.00
Training session 5. Dissemination in social networks						
The concepts dealt with in this section were clear to me.	4.81(0.20)	4.51(0.31)	24.00	0.02^*^	−2.35	0.28
The materials presented in this session have proven useful for my teaching.	4.88(0.13)	4.67(0.31)	30.00	0.06	−1.85	0.17
The complementary information has proven to be useful to me.	4.88(0.13)	4.67(0.31)	30.00	0.06	−1.85	0.17
This session requires more work time.	4.06(1.00)	3.76(0.88)	43.50	0.48	−0.71	0.03

* *p < 0.05*;

** *p < 0.01*.

*Z = Z de Kolmogorov–Smirnov; effect size η^2^*.

With regard to the analysis of the open response answers, 90% of the teachers would not omit anything, although 10% did indicate that there was a lot of information. As regards suggestions for improvement, 90% felt that there should be more practical training whilst 10% would not add anything.

## Discussions

With regard to RQ1, it was found that teachers’ interaction behaviour pattern in LMS differed depending on whether they were either inexperienced or experienced teachers. In general, inexperienced teachers tended to interact more, both during the synchronised and the non-synchronised phase, although differences in interaction did emerge between the two groups. These aspects might be related to the teaching style and the internal expectations of teaching staff towards the training activity. Although all the teachers initially started out with the same interest, certain unseen motivations might be exerting an influence. These aspects are related with the results found in the qualitative analysis of the open questions posed in the initial survey, since differences were found in the level of interest displayed towards the training activity. This indicates the need for further inquiry to analyse teaching styles in e-Learning and b-Learning spaces and which explores in depth teachers’ internal motivation towards teaching in these spaces. Such an analysis would also examine which factors might account for the differences in interaction found in the synchronised and non-synchronised phases of the training activity amongst the various participants. Following García-Peñalvo (2021), the process of digital transformation within the framework of higher education poses a complex challenge which, if it is to be addressed effectively, requires government training proposals and a micro-analytical analysis of how this training is perceived and applied in real situations.

As regards RQ2, no exact correspondence was found between the type of teacher (inexperienced vs. experienced) and the behaviour patterns displayed in LMS during the training phase. This has also been reported in other studies with university students (Sáiz-Manzanares et al., 2021b), indicating that although they may initially be seen as homogeneous groups, differences do exist that are probably linked with motivation towards training and with the style of learning. This aspect has not been dealt with previously but is now a key reference, since digital transformation demands that teaching staff be trained or that their training be brought up to date.

With regard to RQ3, the motivation of the teachers taking part was found to be very high, regardless of whether they were inexperienced or experienced teachers. Differences did, however, emerge in terms of perception amongst the group of experienced teachers in terms of the following aspects: designing avatars to encourage SRL, use of learning analytics systems in LMS, and use of social networks to disseminate content. This might be explained by the generational difference between inexperienced and experienced teachers in that the former might have a greater degree of digital competence in these aspects.

Although achieving consistency in satisfaction is a complex task, structuring training activities in levels in terms of degree of difficulty and skill acquisition might offer one solution to this issue.

### Limitations and Future Lines of Research

The results to emerge should, however, be taken with a certain degree of caution, given the characteristics of the sample (non-random selection, small number of participants and the features thereof—they belong to research groups who are analysing the effectiveness of SRL in the teaching-learning process). Worth highlighting is the need to promote research that applies mixed methods (quantitative and qualitative), since qualitative analysis of the responses to the open questions provides a great deal of information about how the training process is perceived and the needs to be pinpointed. This entails carrying out studies that provide for a microanalytical analysis, which in turn means that ratios must not be too big. Future studies will focus on examining what perception teaching staff who evidence different skill levels and who come from different knowledge areas have of training activities in the digitalisation of teaching. It is important to analyse these training activities, given that the current pandemic triggered by COVID-19 the world over means that teaching staff training, which was formerly carried out face-to-face, must now be done online. Furthermore, the actual training content must respond to what is needed in training teaching skills in digital environments. Further research is thus required into how effective these prove to be. What was previously an optional form of training has now become almost the only form such that, although the study does evidence certain limitations, which are mainly related to the generalisation of the results due to the nature of the sample, it does nevertheless afford the advantage of being a study based on the individual follow-up of participants through various monitoring tools. It also offers a detailed list of the materials and tools applied, which can be consulted in the supplementary material and in the open-access links provided, all of which helps with the replicability of the work.

By way of a summary, Table 6 provides a synopsis of the results found in the studies that served as justification for this work, together with the results to emerge from the work itself.

**Table 6:** Relation between the studies which served as the theoretical basis for the study and the outcomes to emerge from this work.

Previous studies	Results found in this study
Virtual learning environments help SRL (Azevedo et al., 2011, 2015) through various hypermedia resources, such as avatars and serious games. Studies by Kretschmer and Terharen (2019), Nappo et al. (2020), Sáiz-Manzanares et al. (2020), and Van De Weijer et al. (2020) found that the use of gamification enhances cognitive skills and boosts student motivation.	Prior to the training activity, both doctoral teaching staff and students alike displayed an interest in knowing the possible teaching resources that could be used in virtual contexts. They were also eager to know both the basic and the advanced techniques as well as the strategies that could be used in self-regulation. They also expressed a high degree of satisfaction at having taken part in the gamification activities.
Krouska et al. (2020) found that platforms such as Moodle were very useful and easy to use for teaching.	Participant satisfaction in this study with the resources applied in the virtual platform was high, both amongst doctoral teaching staff and students (future university teachers).
A key factor in the development of learning in LMS is to take particular care when devising resources and activities (Jommanop and Mekruksavanich, 2019; Kubik et al., 2021; Sáiz-Manzanares et al., 2021b). SRL is a key skill at all educational levels and can be boosted by developing structured training programmes aimed at teachers, particularly those who are undergoing their training (Dumulescu et al., 2021; Kubik et al., 2021).	Designing a training programme based on self-regulation has helped with the follow-up and analysis of the training process. This method has allowed for an analysis of the learning patterns developed by the participants, for which heat map visualisation techniques have been used. These resources help teachers to see differences in patterns easily and quickly.
The use of meta-tutoring or automatic tutoring resources in LMS aids student-centred learning, fosters student commitment and improves knowledge acquisition (Troussas et al., 2021; Krouska et al., 2021a).	This study reported a high degree of satisfaction with activities that included automatic feedback (e.g., gamification activities). Moreover, in this aspect no significant differences were found between doctoral teachers and students in terms of satisfaction regarding the use of these techniques.
Scheduled synchronous sessions boost collaborative work and assessment systems with feedback on the process (de la Fuente et al., 2021b).	The synchronous stages of the training process have been linked to greater participant access to the Moodle platform vs. less access in asynchronous sessions. Likewise, a difference was found in frequency of access between doctoral teaching staff and students; specifically, in favour of doctoral students with regard to content related to materials for designing rubrics in VLE and the use of Learning Analytics systems in VLE. Likewise, a difference was found in frequency of access between doctoral teaching staff and students; specifically, in favour of doctoral students with regard to content related to materials for designing rubrics in VLE and the use of Learning Analytics systems in VLE, although the motivation for the learning tasks was high in both group.
Teacher training in virtual environments is one of the goals of the 2030 Agenda (Redecker and Punie, 2017; Jarillo et al., 2019; García-Peñalvo, 2021) that has increased as a result of the current COVID-19 pandemic (Redecker and Punie, 2017).	This study offers materials and tools for designing training courses in digital skills for teaching based on self-regulated instruction in virtual environments. This study offers a number of tools for gauging user perception of their satisfaction with learning processes in virtual environments. It also offers serious game materials for implementing automatic feedback on knowledge acquisition. Participant satisfaction with the training process has been evidenced (mean values of four out of five). Greater participation in synchronous than in asynchronous sessions has also been evidenced.
It is important to gain an understanding of what both experienced and inexperienced teachers consider to be the strengths and weaknesses of LMS, which are key references in the current pandemic, particularly in higher education. The use of resources that include automatic personalised feedback procedures is key to enhancing student motivation. The interaction difference between synchronous and asynchronous sessions is evidenced (Collazos et al., in press).	

## Conclusion

Higher education faces a major challenge in terms of teaching in the 21st century. Said challenge, which had already been set out by government authorities in objective 5 of the 2030 Agenda (Redecker and Punie, 2017), has been hastened as a result of the current health crisis brought on by the pandemic (García-Peñalvo, 2021).

### Implications for Teacher Training in Higher Education

In higher education, face-to-face teaching as the only means of teaching is dying out. Current higher education teaching is delivered through e-Learning or b-Learning (García-Peñalvo, 2021). This implies that instructional design must undergo changes compared to the traditional design. These changes are related to the use of the technological and pedagogical resources afforded by student SRL and self-evaluation in order to provide personalised learning (Jommanop and Mekruksavanich, 2019; Kubik et al., 2021; Sáiz-Manzanares et al., 2021b). This entails the use of tools that offer the student intelligent tutoring in LMS (Azevedo et al., 2011, 2015; Taub et al., 2017, 2018; Troussas et al., 2021; Krouska et al., 2021a). In order to achieve this, two key aspects are required; firstly, the functional pedagogical design of LMS that will allow for the inclusion of technology-based resources such as avatars and gamification activities. These environments must also include easy-to-use follow-up tools for tracking student learning behaviour throughout the teaching-learning process (Jommanop and Mekruksavanich, 2019; Sáiz-Manzanares et al., 2021b; e.g. UBUMonitor).

If the challenges facing teaching within the framework of higher education are to be met successfully, it is necessary to design and implement training programmes (de la Fuente et al., 2021a, 2021b) that skill teachers in the use of LMS and the technological tools included in these virtual environments so as to automatically provide SRL and self-evaluation (Dumulescu et al., 2021; Kubik et al., 2021). These training proposals must offer varying levels of difficulty vis-à-vis the acquisition of skills, adapting to each teacher’s training requirements. This prioritisation of skills is related to teachers’ previous knowledge in digitalisation and with the style of teaching they have employed throughout their teaching career. It must be borne in mind that experienced teachers have a background of teaching based on face-to-face interaction. This means that although they may have used innovative teaching techniques, they will have done so in face-to-face contexts. The interaction between teacher and student and between students themselves in these spaces differs enormously from what is found in e-Learning or b-Learning spaces. In the latter, interaction features such as eye contact or comments about what a student or group of students may have done occurs in a much different way to what is found in face-to-face interaction. As a result, in most cases experienced teachers will need to have their digital skills updated. In contrast, inexperienced teachers lack this particular teaching background and normally possess more highly developed digital skills, such that their training should be geared more towards skills related to how to approach teaching in digital environments in terms of applying technologies they are already familiar with (Dumulescu et al., 2021; Kubik et al., 2021).

This study has also shown how interaction in online courses is complex because, although there is a synchronised phase and resources such as forums or chats are available, participant interaction is not always fluid. The same trend can also be seen in e-Learning or b-Learning teaching (Sáiz-Manzanares et al., 2017, 2021a,b). As a result, further research needs to be conducted into how fluent interaction can be improved, whether on the part of the teacher or the student, in order to offset the feeling of loneliness in the net. Achieving this might involve elements related to the use of social networks (de la Fuente et al., 2021b) as a resource for teaching.

In sum, there is still a long way to go before we achieve digital transformation in higher education in terms of the teaching-learning process. In order to accomplish this, further research is required exploring the acquisition of digital skills in university processes. One way of approaching this, in addition to updating teachers’ digital skills, would involve including courses on digitalisation in the curricula of all university degrees. This is the challenge facing those responsible for higher education institutions the world over. In short, teaching staff need to be trained in how to design teaching activities that include SRL processes through ALT, since these resources allow the planning stage to be focused, for example with the use of avatars (Azevedo et al., 2015, Azevedo and Gašević, 2019), the follow-up stage (Azevedo and Gašević, 2019; Hosain et al., 2019; Noroozi et al., 2019), for example using tools similar to UBUMonitor (Sáiz-Manzanares et al., 2021b), and the self-evaluation stage (Kramarski and Michalsky, 2009; Bernardo et al., 2017), for example through the use of gamification activities so as to ultimately enhance motivation towards the goal of learning (Zimmerman, 2008). All of this will aid student development of metacognitive learning strategies when processing information (Carlson, 2003; Rothbart et al., 2011; McClelland et al., 2014; Valadas et al., 2017). This is one of the challenges facing teaching in the 21st century, since the mere use of LMS by no means ensures that deep-seated and reliable learning will be achieved (Yamada and Hirakawa, 2016; Park and Jo, 2017). As a result, training in digital skills, both in terms of their use and design, is the challenge facing educational institutions, particularly those engaged in higher education (Redecker and Punie, 2017; Jarillo et al., 2019; García-Peñalvo and Corell, 2020; García-Peñalvo, 2021).

Summing up, it can be concluded that further studies are needed that delve more deeply into a detailed analysis of instructional processes in digital skills based on self-regulation aimed at teaching staff. There are various resources, such as the use of serious games that include automatic feedback on the learning outcomes that can help with autonomous and personalised learning. Further work should also be carried out on developing resources that help to bridge the gap between participants’ synchronous and asynchronous participation in educational activities. Teacher training, which is quite new in terms of these skills, will prove to be key if these teachers are to later use these tools in their everyday teaching practice.

## Data Availability Statement

The original contributions presented in the study are included in the article/Supplementary Material, and further inquiries can be directed to the corresponding author.

## Ethics Statement

The studies involving human participants were reviewed and approved by University of Burgos Bioethical Committee, No. IR 27/2019, the coordinating university. The patients/participants provided their written informed consent to participate in this study.

## Author Contributions

MS-M and LA: design and initial writing. LM-A and MS-M: review. MS-M, LA, MC and JV-B: final writing. All authors contributed to the article and approved the submitted version.

## Funding

This work was funded through the European “Self-Regulated Learning in SmartArt” Project 2019-1-ES01-KA204-065615.

## Conflict of Interest

The authors declare that the research was conducted in the absence of any commercial or financial relationships that could be construed as a potential conflict of interest.

## Publisher’s Note

All claims expressed in this article are solely those of the authors and do not necessarily represent those of their affiliated organizations, or those of the publisher, the editors and the reviewers. Any product that may be evaluated in this article, or claim that may be made by its manufacturer, is not guaranteed or endorsed by the publisher.
