# Supplementary material for: Teacher Training Effectiveness in Self-Regulation in Virtual Environments
Source: Front Psychol. 2022 Mar 28;13:776806. doi: 10.3389/fpsyg.2022.776806 (PMC9000986; doi:10.3389/fpsyg.2022.776806)
Supplement: Supplementary file 1 [file Data_Sheet_1.pdf]

Supplementary Material

1 Supplementary Figures and Tables

Table S1. Initial survey on prior knowledge of Advanced Learning Technologies (ALT) (Sáiz-Manzanares, 2021).

Items	Rating scale				
1. I believe that the teaching and learning process should be interactive between teacher and student	1	2	3	4	5
2. I have a knowledge of how to design virtual learning platforms.	1	2	3	4	5
3. I have a knowledge of how to design process-oriented feedback	1	2	3	4	5
4. The feedback provided by the teacher on the student's practice should be clear, positive and task-dependent.	1	2	3	4	5
5. I have a knowledge of eye tracking methodology.	1	2	3	4	5
6. I have a knowledge of how to design learning-oriented gamification activities.	1	2	3	4	5
7. I have a knowledge of project dissemination in social networks.	1	2	3	4	5
8. I have previously used gamification experiences as a learning resource.	1	2	3	4	5
9. I have used the Alexa skill to monitor learning activities.	1	2	3	4	5
10. What are your expectations vis-à-vis the training activity?	1	2	3	4	5
11. What would you like to learn in the training activity?	1	2	3	4	5

Table S2. Guide to the training proposal on ALT.

This learning activity is designed to be eminently practical.
The main objectives are for participants to learn what a Virtual Learning Environment (VLE) is, how to design it in an intelligent classroom and how to measure the interaction results.
Methodology: The work proposal is centred around the project-based learning method. Participants work in small groups that can be made up of members from each partner. They draw up a design project for a virtual intelligent classroom for specific learning content and which they themselves choose. This project should contain: a design of the learning activities from a self-regulated perspective using process-oriented gamification elements and feedback.

First session. What a virtual learning environment is and how it is used.

Total time: 5 hours.

First part (I): Information on the working methodology of the training activity: project based learning.

Time: 1 hour presentation.

Second part (II): How to work in a VLE.

Time: 1 hour presentation.
Break: half an hour.

Third part: collaborative work

Time: 2.5 hours of collaborative work on the proposals put forward in the first part of the training session.

Second session. How to design learning materials using an avatar. Proposals in virtual environments.

Total time: 5 hours.

First part (I): Designing learning materials based on gamification.

Time: 1 hour presentation.

Second part (II): How to design learning materials using an avatar. Proposals for designing games in virtual environments.

Time: 1 hour presentation.

Break: half an hour.

Third part: Collaborative work

Time: 2.5 hours of collaborative work on the proposals put forward in the first part of the training session.

Third session. How to design process-oriented assessment rubrics in a virtual intelligent classroom.

Time: 5 hours.

First part (I): Designing rubrics in a SmartArt project

Time: 1 hour presentation.

Second part (II): How to design process-oriented assessment rubrics in a virtual intelligent classroom.

Time: 1 hour presentation.

Break: half an hour.

Third part (III): Collaborative work

Time: 2.5 hours of collaborative work on the proposals put forward in the first part of the training session.

Fourth session. Assessment methods for the learning process in virtual learning environments: introduction to eye tracking technology.

Time: 5 hours.

First part (I): Assessment methods for the learning process in virtual learning environments: introduction to *eye tracking* technology.

Time: 1 hour presentation.

Second part (II): An example of data processing.

Time: 1 hour presentation.

Break: half an hour.

Third part (III): Collaborative work

Time: 2.5 hours collaborative work on the proposals put forward in the first part of the training session.

Fifth session. How to spread our project in social networks and through other means.

Time: 5 hours.

First part (I): Social networks as a means of spreading teaching innovation projects.

Time: 1 hour presentation.

Second part (II): Dissemination through different means of communication.

Time: 1 hour presentation.

Break: half an hour.

Third part (III): Collaborative work

Time: 2.5 hours collaborative work on the proposals put forward in the first part of the training session.

Table S3. Gamification activities used in the proposed training (Sáiz-Manzanares, 2021).

Gamification activity 1.	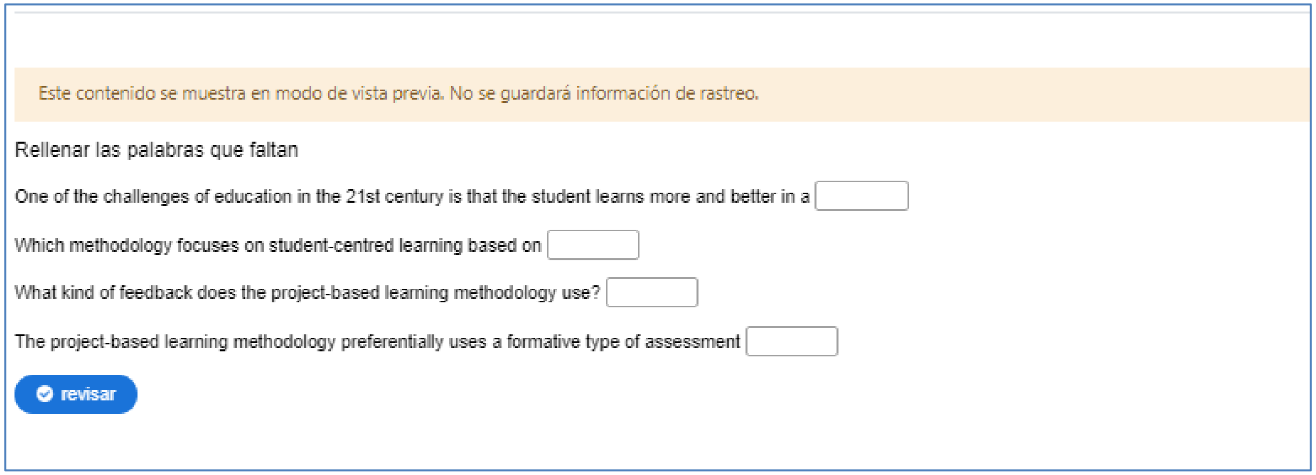
Gamification activity 2.	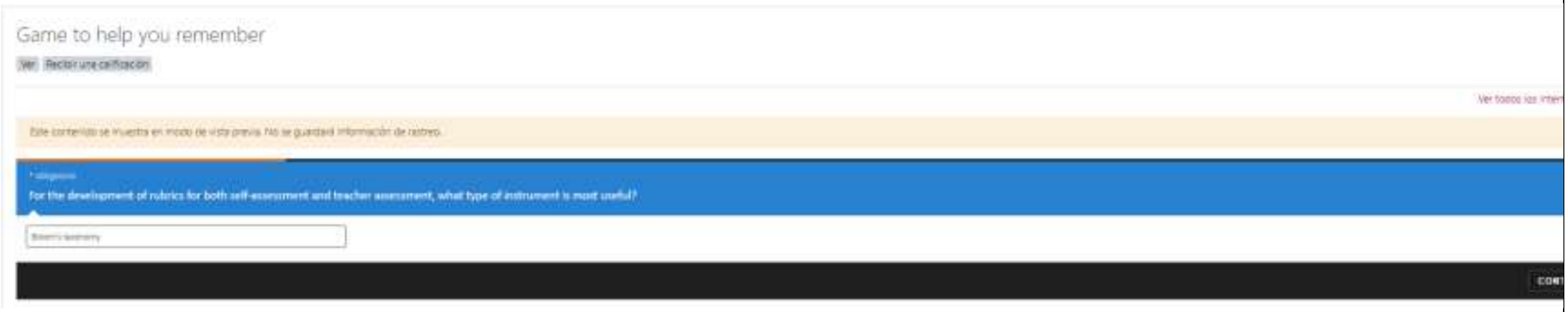

Gamification activity 3.

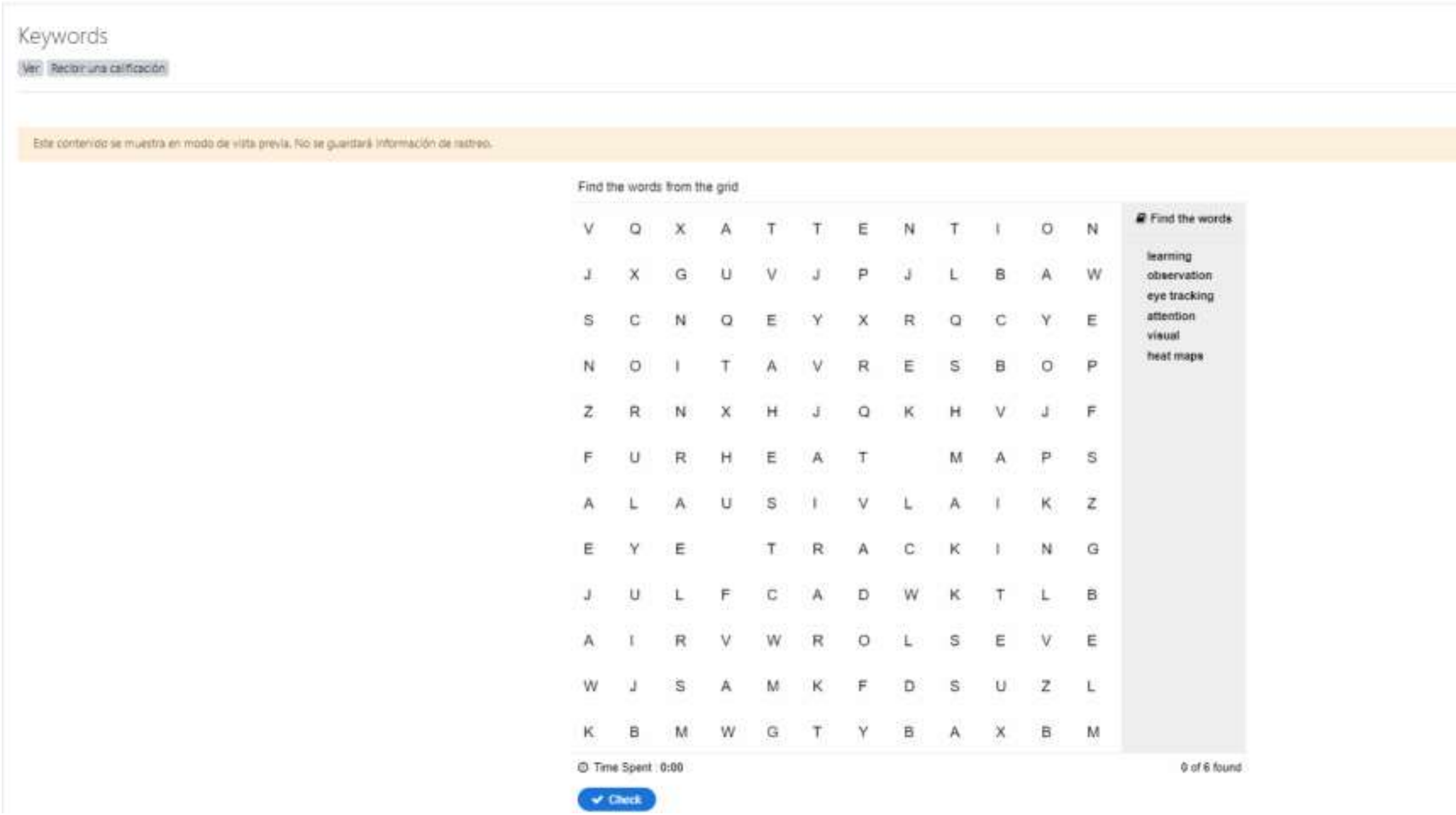

Gamification activity 4.	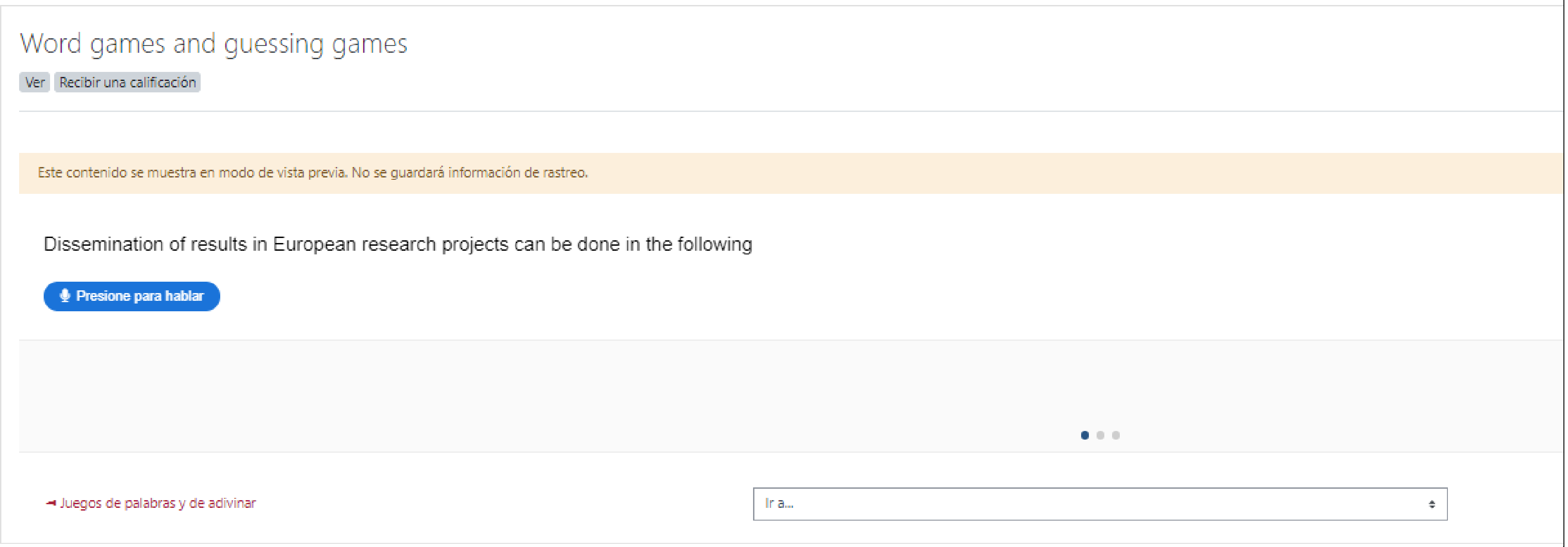

Table S4. Satisfaction survey with the synchronised sessions (Sáiz-Manzanares, 2021).

Items	Rating scale				
1. The concepts dealt with in this section were clear to me.	1	2	3	4	5
2. The materials presented in this block have proven useful for my teaching.	1	2	3	4	5
3. The complementary information has proven useful to me.	1	2	3	4	5
4. This session requires more work time.	1	2	3	4	5
5. Indicate which aspects need to be extended in this part of the course.	1	2	3	4	5
6. Indicate which aspects need to be removed from this part of the course.	1	2	3	4	5
7. Suggestions for improvement.	1	2	3	4	5

Table S5. Satisfaction survey with the training activity.

Items	Rating scale				
1. Communication with the meeting coordinator.	1	2	3	4	5
2. Learning Activity Agenda.	1	2	3	4	5
3. Presentation on the ongoing progress by the project coordinator.	1	2	3	4	5
4. Time management.	1	2	3	4	5
5. Atmosphere and communication among attendees.	1	2	3	4	5
6. Would you be interested in using the proposed tools in your job?	1	2	3	4	5
7. Do you consider that the tools presented are easy to use when teaching?	1	2	3	4	5
8. Do you consider that specific training is required to use the tools presented?	1	2	3	4	5
9. Would you like to spread the proposed tools among your colleagues?	1	2	3	4	5
10. Quality of the virtual environment in which the training has been carried out.	1	2	3	4	5
11. The gamification activities have made it easier for me to understand the concepts.	1	2	3	4	5
12. Their satisfaction with the duration of the training activity is.	1	2	3	4	5
13. Which of the gamification materials have you found most useful for understanding the concepts	1	2	3	4	5
14. What elements would you introduce or increase in gamification materials?	1	2	3	4	5
